# LiDAR‐guided Archaeological Survey of a Mediterranean Landscape: Lessons from the Ancient Greek Polis of Kolophon (Ionia, Western Anatolia)

**DOI:** 10.1002/arp.1572

**Published:** 2017-04-25

**Authors:** Benedikt Grammer, Erich Draganits, Martin Gretscher, Ulrike Muss

**Affiliations:** ^1^ Department of Prehistory and Historical Archaeology University of Vienna Franz‐Klein‐Gasse 1 1190 Vienna Austria; ^2^ Department of Geodynamics and Sedimentology University of Vienna UZA II, Althanstrasse 14 1090 Vienna Austria; ^3^ LBI for Archaeological Prospection Hohe Warte 38 1190 Vienna Austria; ^4^ Department of Classical Archaeology University of Vienna Franz‐Klein Gasse 1 1190 Vienna Austria

**Keywords:** Airborne laser scanning (ALS), light detection and ranging (LiDAR), archaeological survey, Ionia, Turkey, remote sensing

## Abstract

In 2013, an airborne laser scan survey was conducted in the territory of the Ionian city of Kolophon near the western coast of modern Turkey as part of an archaeological survey project carried out by the Mimar Sinan University of Istanbul (Turkey) and the University of Vienna (Austria). Several light detection and ranging (LiDAR) studies have been carried out in the temperate climate zones of Europe, but only a few in Mediterranean landscapes. Our study is based on the first LiDAR survey carried out for an archaeological purpose in Turkey and one of the first in the Mediterranean that have been planned, measured and filtered especially for archaeological research questions. The interpretation of LiDAR data combined with ground‐observations proved extremely useful for the detection and documentation of archaeological remains below Mediterranean evergreen vegetation and dense maquis. This article deals with the methodological aspects of interpreting LiDAR data, using the Kolophon data as a case study. We offer a discussion of the strengths and limitations of LiDAR as an archaeological remote sensing method and suggest a best practice model for interpreting LiDAR data in a Mediterranean context. © 2017 The Authors. *Archaeological Prospection* published by John Wiley & Sons Ltd.

## Introduction

Light detection and ranging (LiDAR) provides high‐resolution digital terrain models (DTMs) which are becoming more and more essential for archaeological prospection (Crutchley and Crow, 2010; Chase *et al.*, 2012; Opitz and Cowley, 2013a; Bernardini *et al*., 2015). Due to its capacity to measure even subtle topographic features in high detail and its ability to remove vegetation by filtering processes, it is becoming the method of choice for the archaeological study of forested areas (Doneus *et al.*, 2008). Compared to agricultural areas, the micro‐topography is usually better preserved and can be visualized with the last echo of the LiDAR reflections (Ackermann, 1999; Wehr and Lohr, 1999; Poirer *et al.*, 2013).

The archaeological interpretation of LiDAR data is primarily a method for discovering surface monuments and an aid in contextualizing them in their physical environment. LiDAR offers a non‐selective and thus ‘messy’ (Mlekuž, 2013a) perspective of the physical landscape in almost overwhelming detail. A growing number of case studies using LiDAR data exist, commonly with very remarkable results (e.g. Chase *et al.*, 2011; Bernardini *et al.*, 2015). A recent compendium by Opitz and Cowley (2013a) summarizes various aspects of archaeological LiDAR data interpretation and contains efforts to contextualize it in the theoretical framework of landscape archaeology (e.g. Mlekuž, 2013a; Opitz and Cowley, 2013b). However, so far only a few articles have yet to explicitly describe the methodology that was used for the interpretation of LiDAR data in a specific archaeological research project (e.g. Doneus and Kühtreiber, 2013). As every landscape and archaeological heritage has unique properties that require different methodological approaches, there are no ready‐made recipes or procedures for the interpretation of LiDAR data (Mlekuž, 2013b, p. 127). Yet, some degree of consent and methodological explanation about the transformation of high‐resolution topographic data into an archaeological interpretation is highly desirable.

**Figure 1:**
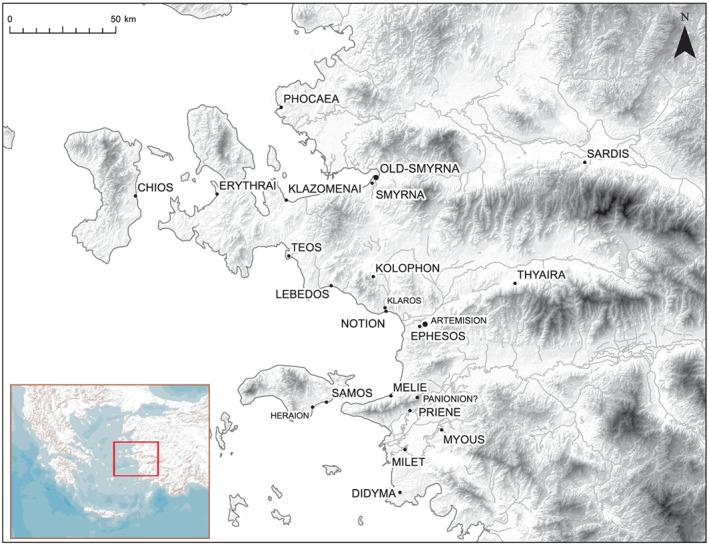
The Ionian Dodekapolis with the extra‐urban sanctuaries and their Lydian neighbours [Topographic data derived from EU‐DEM (http://land.copernicus.eu/in‐situ/eu‐dem‐derived‐products/eu‐dem) and Open Street Map (https://www.openstreetmap.org), place locations derived from Pleiades project (http://pleiades.stoa.org) with minor corrections by author]. [Colour figure can be viewed at wileyonlinelibrary.com]

**Figure 2:**
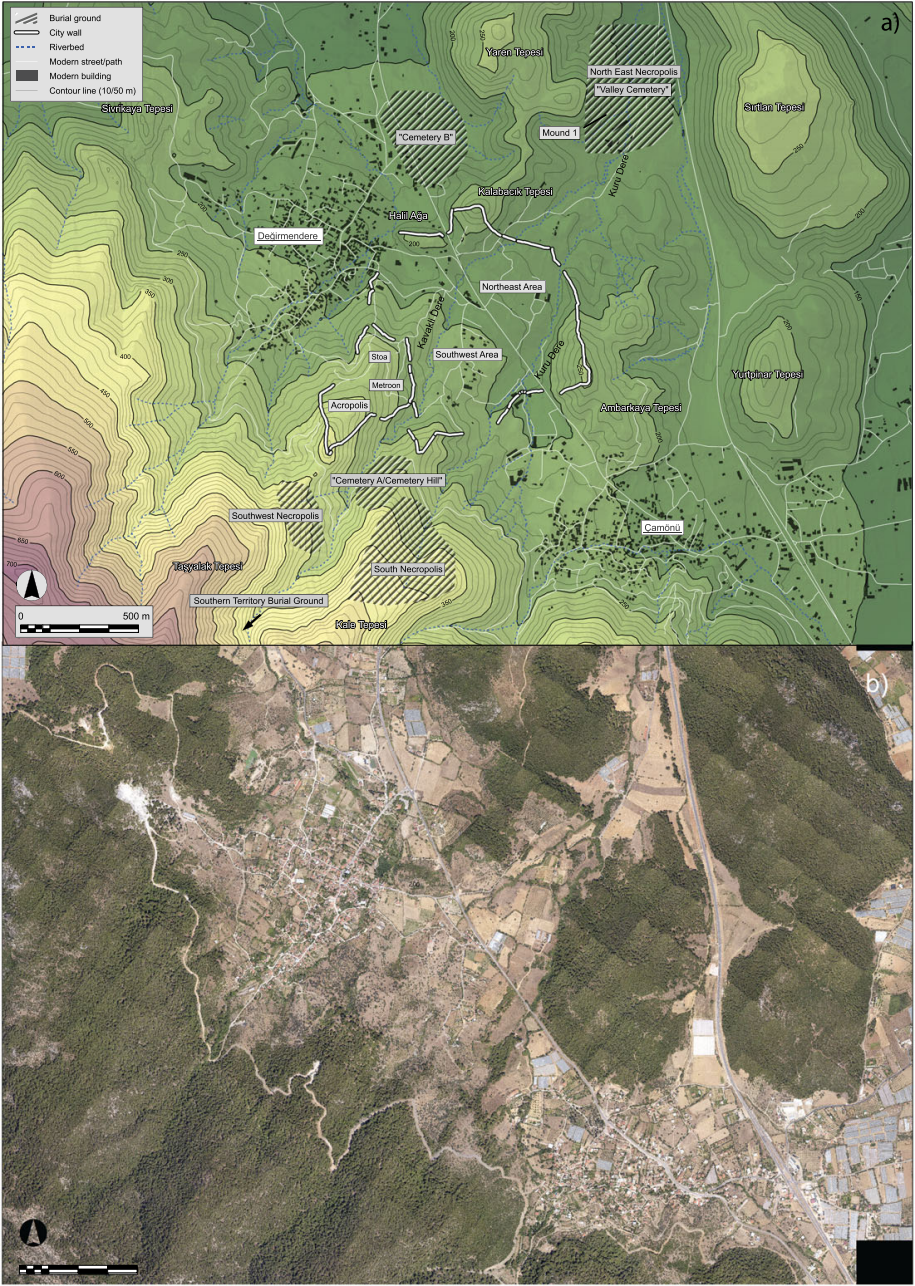
(a) Kolophon with zones of archaeological interest and modern topography. Topographic data derived from LiDAR scan, height in reference to GRS80 ellipsoid; (b) orthophoto mosaic of same area. [Colour figure can be viewed at wileyonlinelibrary.com]

Usually, LiDAR measurements are carried out in a context of land surveying and flood prevention (Bales *et al.*, 2007). LiDAR surveys conducted especially for archaeological research are rare, especially in the Mediterranean (Poirer *et al.*, 2013; Doneus *et al.*, 2015). Most examples of LiDAR‐based archaeological surveys have taken place in Middle and Northern Europe, covered by mainly deciduous vegetation. Therefore, there is a pronounced need for more experiences with LiDAR surveys in the Mediterranean with its rich archaeological heritage, which in many places are covered by typical evergreen vegetation and dense maquis (Campana *et al*., 2007; Doneus *et al.*, 2015). Our study is based on the – to the best of our knowledge – first LiDAR survey carried out for an archaeological purpose in Turkey. It was conducted as part of a project with the aim of documenting the material remains and reconstructing the urban development of the Ionian *polis* of Kolophon (Figures 1 and 2; Gassner *et al*., 2017).

**Figure 3:**
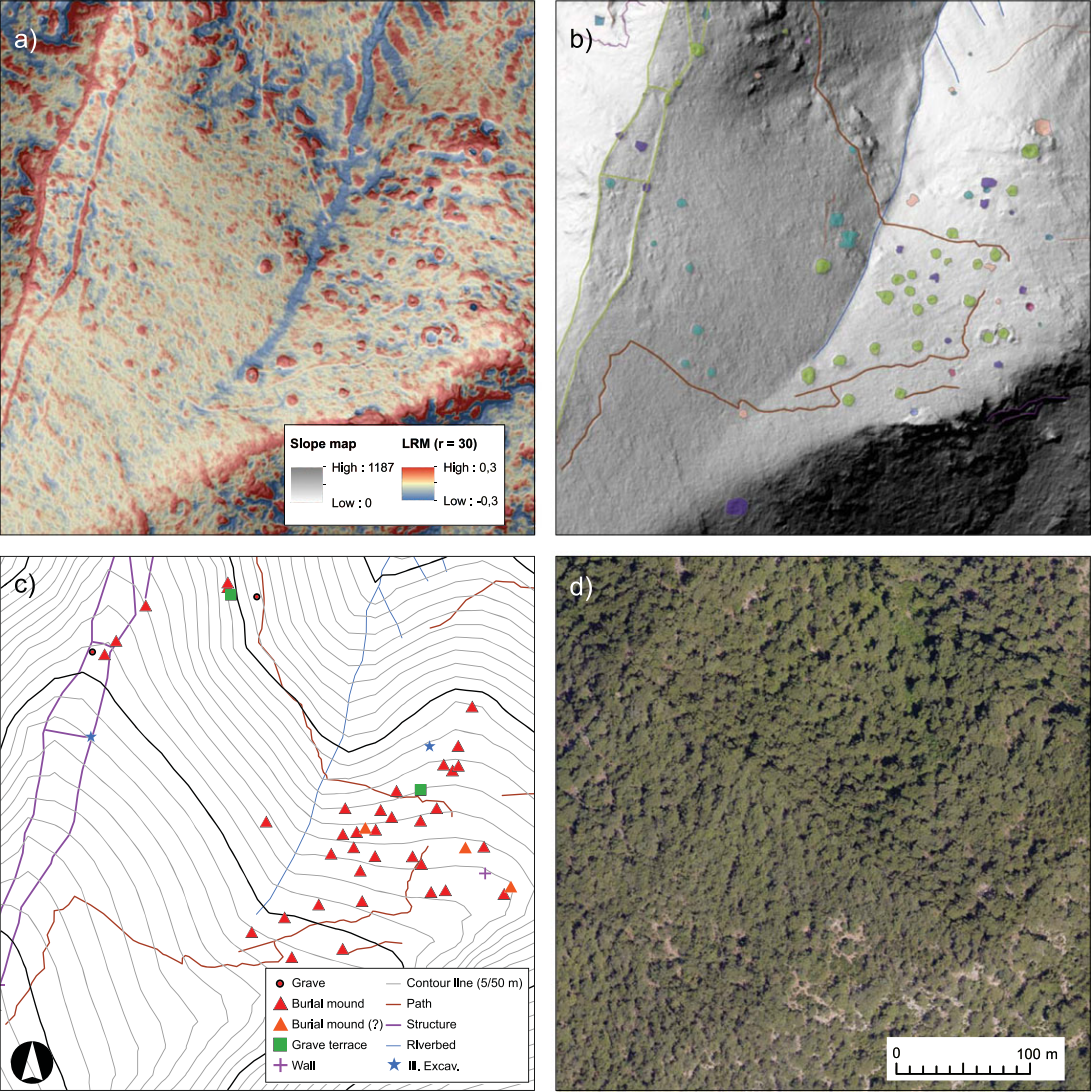
South necropolis of Kolophon on Taşyalak Tepesi. (a) Local relief model [radius (*r*) = 30; histogram stretch = ˗0.3/0.3], combined with a slope map (50% opacity, histogram stretch = standard deviations). (b) Hillshade [azimuth (*a*) = 315, elevation angle (*e*) = 35, histogram stretch = standard deviations] with traced features of the remote sensing interpretation. (c) Topographic map with archaeological objects after ground observation. (d) Orthophoto of the area. [Colour figure can be viewed at wileyonlinelibrary.com]

LiDAR interpretation in archaeology is only some 15 years old and its methodological and theoretical background is still under discussion; a central concern is how to derive archaeological information from LiDAR data (Opitz and Cowley, 2013b, pp. 4–6; Figure 3). In the present article we use the experiences of handling LiDAR data in the Kolophon survey to offer a detailed description, analysis and discussion of the entire process of data handling, processing, visualization, and archaeological interpretation. Our intention is to make the interpretation process reproducible.

At the beginning of any interpretation of LiDAR data the research goal must be defined. Further steps will need to be determined in relation to this research question. The general workflow (cf. Doneus and Kühtreiber, 2013, pp. 33–35) entails:

- assessing the geological, archaeological and modern setting of the project area
- gathering and processing of data
- evaluation of the metadata
- data visualization
- interpretative mapping
- ground‐observation
- interpretation and contextualization of the results

This is not a strict step‐by‐step process, since the various steps are related to each other and are conducted in an integrative and sometimes iterative fashion (Doneus and Kühtreiber, 2013, p. 33).

## Historical, archaeological and geological setting of Kolophon

Kolophon is an Ionian city located near the western coast of Turkey, 35 km south of modern Izmir (Figure 1). In ancient times the city was part of the league of 12 Ionian cities and was notably the only one not situated directly at the coast. The city can be reached from the coast after some 16 km along the narrow Ahmetbeyli (ancient Ales) valley. During the Geometric period, the distance to the coast might have been about 1.5 km shorter (Doğan, 2013). The city was closely aligned with the Apollon sanctuary of Klaros and later with the port city of Notion (in general see Bürchner, 1921, p. 1017–1018; Rubinstein and Greaves, 2004). The city was known for its wealth and the quality of its cavalry forces in the Archaic period, but after a series of violent struggles with the Lydian Empire, its importance diminished. Kolophon played an active, albeit minor role in the Persian wars, and was conquered by Lysimachus' forces in Hellenistic times. After an unsuccessful attempt at resistance, the polis was dissolved and its population transferred to Ephesos.

Archaeological research in the city was limited (Figure 2). The city was identified and mapped by Schuchhardt (1886); the American School of Classical Studies at Athens (ASCSA) and the Fogg Art Museum of Harvard University carried out two excavation campaigns in 1922 and 1925 (Goldman, 1923; Holland, 1944). Afterwards research was limited to mostly unpublished rescue excavations by the Museum of Izmir (Şahin, 2008). In 2010 a Turkish–Austrian survey resumed archaeological research at Kolophon (Bruns‐Özgan *et al.*, 2011; Gassner *et al*., 2017).

In terms of geology, the study area is situated on the north‐eastern slope of the Karacadağ, facing the Cumaovası Basin. Kolophon's urban area is located at an altitude range between 140 m to 327 m above sea level (a.s.l.).

Lithologically, Kolophon/Değirmendere is mainly situated in the Bornova Flysch Zone (BFZ), which is tectonically thrust on top of the Menderes Massif (Jolivet *et al.*, 2013; Gessner *et al.*, 2011; Okay *et al.*, 2012). The BFZ is a virtually unmetamorphosed olistostrome/mélange and comprises late Cretaceous to lower Paleocene shale, sandstone and rarely conglomerate, deposited by deep‐water marine gravity flows (Okay and Altıner, 2007). These clastic sediments contain randomly distributed metre‐ to kilometre‐sized olistolith blocks. These blocks comprise Mesozoic shallow marine limestone and ophiolithic blocks from an oceanic lithosphere (Okay *et al.*, 2012). In contrast, the Cumaovası Basin in the northern part of the study area comprises lower Miocene to recent fluvial/lacustrine clastic sediment and lacustrine carbonates with some volcanic layers (Uzel and Sözbilir, 2008; Uzel *et al.*, 2013).

The weather station of the Izmir Adnan Menderes Airport (125 m a.s.l., 20 km north‐northeast of the study area) reports in the period 1989–2014 an annual average temperature of 16.8°C (15.5–18.0°C) and annual average precipitation of 746 mm (406–1133 mm) (TuTiempo, 2015). Although the annual precipitation is not very high, the very uneven distribution of rain, concentrated in the autumn and spring months result in episodic intensive showers, which contribute to erosion by surface run‐off.

The geological setting and geomorphological processes outlined earlier strongly control the topography, micro‐topography and vegetation and have a direct impact on the terrain surface visualized in the LiDAR data. Consequently, they exert a strong influence on the visibility and preservation of archaeological features. Basic knowledge about the local geological setting and geomorphological processes is an important prerequisite for the proper interpretation of remote sensing data (e.g. Draganits *et al.*, 2015, pp. 96–110).

The alluvial lowlands are well suited for intensive agricultural use and due to millennia of intensive plowing, hardly any archaeological features are visible in LiDAR data and aerial photography. In contrast, archaeological features are much better preserved in the forested areas, which are mainly found in the hilly area consisting of the BFZ. Shale and sandstone areas of the BFZ as well as the young basin fill of the Cumaovası Basin generally show smooth surfaces formed by surface run‐off, which support high visibility of anthropogenic structures. However, in many areas interpretation is complicated by the existence of the randomly distributed limestone olistoliths of various sizes mentioned earlier, which can be confused with burial mounds in the LiDAR data. Thick dolomite successions above the BFZ are dominated by underground drainage. They occasionally show open joints and rock falls in steep areas, both related to slope instabilities.

At present, most of the hills surrounding ancient Kolophon are quite densely covered by forests, often with almost impenetrable understory (Figure 2). These areas are too steep and/or rocky for agriculture, but are quite well suited for a pastoral economy, for which they are used up to the present day. According to excavation photographs from 1922, archived in the ASCSA, these hills were covered by maquis, but were far less forested back then (http://www.ascsa.edu.gr/index.php/archives/Kolophon‐1922‐photos).

## Metadata of the LiDAR scan

In July 2013 the Austrian company Airborne Technologies conducted an airborne laser scan. It covered an area of 21 km^2^ with an altitude range from 65 to 700 m (a.s.l.). The average point density was 12 points per m^2^, with a relative position accuracy better than ±7 cm and relative height accuracy on plane surfaces better than ±7 cm. The measured point cloud was filtered to create a digital surface model (DSM) from the first echo as well as a DTM from the last echo.

The evaluation of the DTM requires an understanding of the relatively complex technical details of data processing and filtering (Doneus and Briese, 2011; Doneus, 2013a, pp. 213–216; Opitz, 2013, pp. 20–28). Depending on the filtering and interpolation applied, the same raw data can generate different surfaces (Doneus, 2013a, p. 216). In the case of the Kolophon project the processing was carried out by Airborne Technologies within Turkey. The raw data was not accessible for legal reasons. It was therefore not possible to apply different filtering algorithms and compare the resulting DTMs. Evaluation of the scan data was limited to comparing the first‐echo DSM with the filtered DTM, and matching both with known, topographical features observed on the ground. At the same time the LiDAR measurements were made, aerial images were acquired and orthorectified using the TerraPhoto software.

## LiDAR data interpretation

### Visualizations of LiDAR data

LiDAR data can be transformed into a DTM and visualized in several different ways. It is common experience that no single visualization is sufficient for good results in interpreting LiDAR data (Kokalj *et al.*, 2013, pp. 102–103). Bennett *et al.* (2012) interpreted LiDAR scan data in different visualizations, including slope, aspect, principal component analysis (PCA), local relief model (LRM) and sky‐view factor (SVF). Only less than 77% of the recorded features from the interpretation of combined datasets were found in single visualizations (Bennett *et al.*, 2012, pp. 42–44). A large variety of visualizations have been suggested for archaeological prospection (Challis *et al.*, 2011; Bennett *et al.*, 2012; Doneus and Kühtreiber, 2013; Kokalj *et al.*, 2013). The sheer amount makes it advisable to consider beforehand their specific properties and especially the time consumption of their creation and interpretation. The choice will depend on the scale and the primary research interest, e.g. the prospection of new features, the contextualization of known sites, or the investigation of environmental variables.

In the case of the Kolophon survey project, the aim was to locate and map unknown features within the mostly wooded hills surrounding the city. An archaeological evaluation of the LiDAR interpretations on the ground, i.e. a ground‐observation, was planned as an integral part of the survey campaign (Figure 3). At the beginning, visualizations were used that offer an intuitive reading of the height data (hillshade, slope), followed by a more abstract visualization (LRM) for the detection of smaller and less clearly defined features in the advanced stages of interpretation, and finally, visualizations that enable the clearest outlining of identified features for a detailed documentation (positive/negative openness). Visualizations were used concurrently. The interpreter would frequently switch back and forth between them to examine possible features in various data representations and the aerial photography.

The LRM visualization was created with the LiVT software, developed by Ralf Hesse (http://sourceforge.net/projects/livt/), all other visualizations with the RVT 1.1 toolbox developed by Zakšek *et al.* (2011) (http://iaps.zrc‐sazu.si/en/rvt#v).

### Interpretative mapping

**Figure 4:**
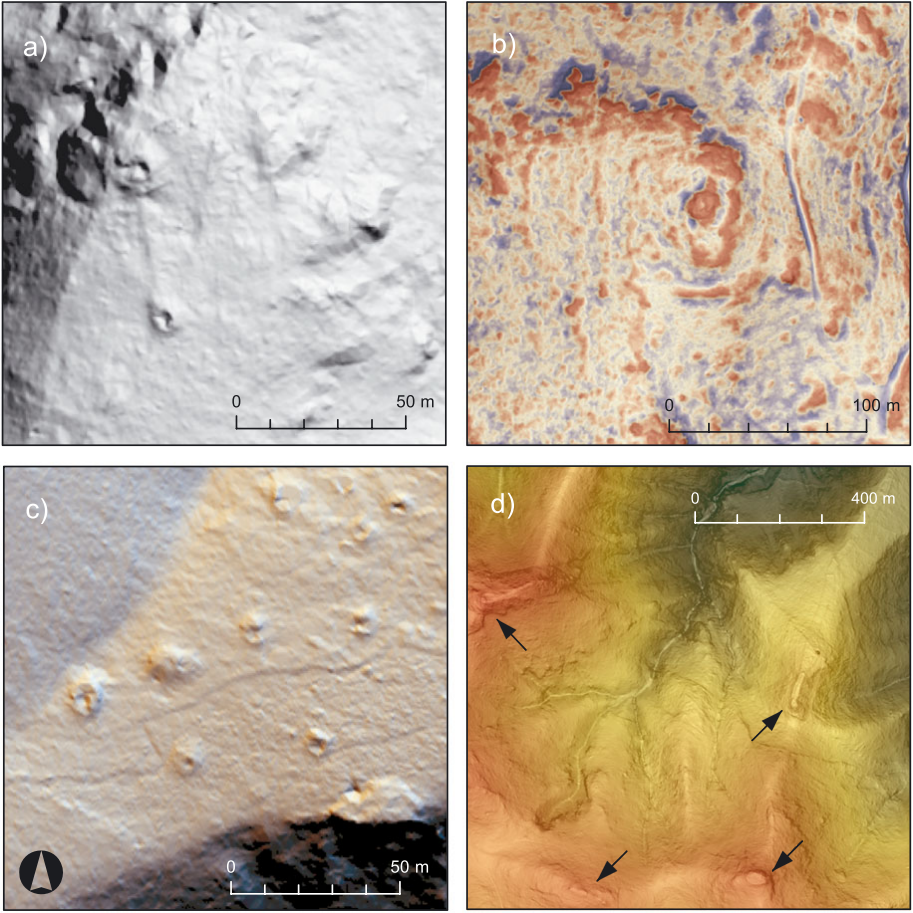
Criteria for interpreting LiDAR features. (a) Hillshade (*a* = 315, *e* = 35, histogram stretch = standard deviations) with burial mounds among rock outcrops. Other than their general size and shape, the trenches of illicit excavations are clear arguments for identifying the features as burial mounds. (b) Local relief model (LRM) (*r* = 30, histogram stretch = ˗0.3/0.3) and slope map (50% opacity). The rectangular shape of the elevations is an indication for anthropogenic structures, most likely terrace or fortification walls. At the top of the terraced hilltop is a structure of unclear function. (c) Multishade [directions (*d*) = 16, histogram stretch = standard deviations]. Burial mounds are placed regularly at intervals of 15 to 20 m. (d) Slope map (histogram stretch = standard deviations) with superimposed terrain map of hilltops in southern territory of Kolophon. At all hilltops visible in the picture anthropogenic structures were found, most likely of different functions. [Colour figure can be viewed at wileyonlinelibrary.com]

The interpretation process consists of the identification and archaeological interpretation of surface features (Doneus and Kühtreiber, 2013, p. 34; Figure 3). This is based on the recognition of contrasts in the visualization of the scan data, which will usually represent changes of surface height. These contrasts are recognized as shapes, which are then related to archaeological objects they might represent (Doneus, 2013a, pp. 135, 190–196). The interpretation of LiDAR data can be guided by criteria similar to those used in remote sensing of satellite imagery and aerial photography (e.g. Lasaponara and Masini, 2012, pp. 7–9). The general aspects of interpretation are well understood and explained (e.g. Wilson, 1982; Brophy and Cowley, 2005; Palmer, 2013; Doneus, 2013a, pp. 190–205), but they are rarely made explicit within archaeological studies. This makes it difficult for readers to evaluate the results, especially if they lack experience in remote sensing. Therefore, in our study we tried to describe the criteria used for the interpretation of each individual feature. Namely these are the form‐oriented factors of *tone* and *brightness*, *shape*, as well as the contextual factors of *pattern*, *association* and *setting* (Figure 4). These criteria provided the guidelines for describing and thereby objectifying the interpretation process to the best extent possible.

Any possible feature was first recognized through differences in *tone* and *brightness*. Both provided different information depending on the visualization and the display properties (Figure 4a). Variations in tone and brightness also showed the surface condition of features, which was crucial for the differentiation between natural and anthropogenic features.

The recognized *shape* of a feature provided the primary argument for its classification (Figure 4b). The recognition of shapes was strongly influenced by archaeological foreknowledge and an understanding of modern agricultural and building processes. Known archaeological features that had already been mapped provided a framework for interpretation, as did general publications of LiDAR features (e.g. Mlekuž, 2013b, p. 121, figure 6.2). Physical parameters of the feature's size such as length, diameter, area and height were measured and compared with other features of the survey and the foreknowledge of the interpreter.

*Texture* refers to the appearance of features in aerial photography or satellite imagery. The combined interpretation of LiDAR data and orthophotos was extremely helpful, as it allowed the modern alterations of the terrain to be easily identified.

*Pattern* and *association* are related criteria and refer to the spatial relationships of features. While single burial mounds were hard to differentiate from geological features like rock outcrops, a cluster of such elevations in a regular *pattern* with regular distances from one another may represent good arguments for a burial ground (Figure 4c). Similarly, a spatial *association* of suspected features with known objects was a major factor of interpretation. The mere presence of known archaeological objects or traces of human activity, such as pathways, will almost inevitably lead to an increased probability of identifying other features in their vicinity. *Association* was an influential, but often misleading factor. It needs to be considered carefully to avoid circular arguments and the introduction of interpretative bias.

Another contextual criterion is *setting*, which refers to the location of features in relation to environmental factors such as absolute height, prominence, slope, the presence of water, etc. Examples include hilltops, which may attract fortifications or burial monuments (Figure 4d). Location factors can vary widely according to regional, chronological and cultural differences, successfully including them in the interpretation therefore rests on the foreknowledge of the interpreter.

Interpretation should usually move from a simple to a more complex level (Girard and Girard, 2003, p. 97). For the general reconnaissance, mostly hillshades and slope maps were used to identify larger, easily recognizable features. The detailed interpretation commonly relied on the combination of an LRM and a slope map. Applying the interpretation criteria outlined earlier, the features were located and examined in multiple visualizations and the aerial photography before they were described and categorized.

It is noteworthy that the LiDAR interpretation included natural and modern features, such as dry riverbeds, open rock joints or modern landscaping and building activity, both because of their archaeological or palaeo‐environmental relevance and to remove them as visual distractions.

**Figure 5:**
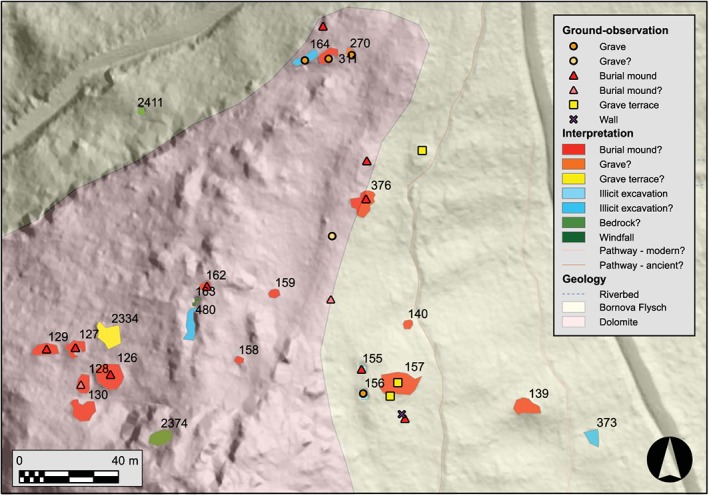
South necropolis of Kolophon on Taşyalak Tepesi. LiDAR hillshade (*a* = 45, *e* = 35; histogram stretch = standard deviations) with geological boundaries, LiDAR interpretation, and results of ground‐proofing. The feature numbers refer to the entries in Table 1. [Colour figure can be viewed at wileyonlinelibrary.com]

To objectify the interpretation processes, the outline of features was traced in a geographical information system (GIS) environment and their properties documented in a geodatabase (Figure 5; Table 1), which included:

- Geometric properties (area, diameter, length, relative height to surrounding area).
- Interpretation metadata (author, time, visualization).
- Description (e.g. depression, elevation, ridge, etc., verbal description)
- Interpretation rationale (criteria and reason for interpretation)
- Interpretation result (e.g. burial mound, wall, terrace, path, etc.).

**Table 1:** Excerpt from the LiDAR interpretation geodatabase in reference to objects in Figure 5.

ID	Ty	Ar	VIS	Int	Description	Rationale	Object
126	E	93,05	LRM	BM?	round regular elevation with depression in the middle	shape, size, tone suggest robbed burial mound; in close proximity to several similar features, likely burial mound, part of Classical necropolis?	TSW10–30
127	E	49,81	LRM	BM?	round regular elevation with depression in the middle	shape, size, tone suggest robbed burial mound; in close proximity to several similar features, likely burial mound, part of Classical necropolis?	TSW10–31
128	E	47,50	LRM	BM?	round regular elevation with depression in the middle	shape, size, tone suggest robbed burial mound; in close proximity to several similar features, likely burial mound, part of Classical necropolis?	TSW10–33
129	E	43,39	LRM	BM?	round regular elevation with depression in the middle	shape, size, tone suggest robbed burial mound; in close proximity to several similar features, likely burial mound, part of Classical necropolis?	TSW10–32
130	E	51,07	LRM	BM?	round regular elevation with depression in the middle	shape, size, tone suggest robbed burial mound; in close proximity to several similar features, likely burial mound, part of Classical necropolis?	NA
139	E	66,62	LRM	BM?	round regular elevation with depression	shape, size, tone suggest robbed/eroded burial mound, proximity to pathway/other features, but very low and undefined outline,	Windfall
140	E	12,07	LRM	BM?	round regular elevation	barely visible burial mound?	NA
155	D	12,89	HS	IE	small depression, regular outline	likely series of illicit excavation within burial ground, steep contrast change to surrounding area	TSW10–37
156	D	9,93	HS	IE	small depression, regular, well defined outline	likely series of illicit excavation within burial ground, steep contrast change to surrounding area	TSW10–38
157	E	141,21	HS	BM?	round, large, irregular elevation	possibly low, heavily eroded burial mound, depression in middle suggest illicit excavation in distant past?	TSW10–39
158	E	9,12	HS, LRM	BM?	round, regular elevation	small, but regular size and marked height change in contrast to even geology → likely burial mound	NA
159	E	13,35	HS, LRM	BM?	round, regular elevation	small, but regular size and marked height change in contrast to even geology → likely burial mound	NA
162	D	13,71	LRM	BM?	round, relatively deep depression	well defined, deep depression suggest recent digging activity at burial mound, proximity to other features	TSW10–29
163	D	11,63	HS	WF	small depression	possible illicit excavation, but aerial photography → windfall	NA
164	D	22,58	HS	IE?	long, rectangular, depression	length of approximately 10 m and regular outline → possible excavation trench? or illicit excavation? close proximity to other features	TSW10–25
270	D	13,36	HS	G?	small, round, irregular depression	Possibly robbed grave, series of contrast changes in proximity suggest multiple illicit excavations	TSW10–27
311	E	33,42	HS	BM?	slight, irregular elevation with adjunct depression	possibly small, robbed burial mound, series of contrast changes suggest multiple illicit excavations	TSW10–26
373	D	27,06	HS	IE?	large, regularly defined depression	almost rectangular depression, possible illicit excavation, but relatively removed from other features	—
376	E	108,99	HS	BM?	irregular elevation	rugged surface and very irregular outline suggest bedrock, but close proximity to other features and necropolis, should be checked during ground‐observation	TSW10–35
480	D	50,48	HS	IE?	long, deep depression	rugged surface, probably part of geology, but size and depth could also be illicit excavation, check during ground‐observation	NA
2334	E	70,87	HS	GT?	irregular elevation with depression in middle	irregular shape, could be robbed/eroded grave terrace, many possible burial mounds in vicinity	NA
2374	D	45,66	HS	R?	irregular depression	proximity to other features, but rugged surface, irregular, likely bedrock	NA
2411	D	5,92	LRM	R?	round depression,	relatively large, but not deep depression, part of similar irregular surrounding geology, more likely natural feature than illicit excavation	NA

Note: Ty: general type of feature, elevation or depression; Ar: area of polygon in m2; VIS: visualization in which feature was identified; Int: interpretation type, BM: burial mound; G: grave; GT: grave terrace; IE: illicit excavation, WF: windfall; R: bedrock; Object: refers to the type of feature as determined during ground‐observation.

Uncertainty in the interpretation was simply marked with a question mark. The detailed description of feature properties and settings can be considered a part of the interpretation. A systematic description served as the starting point for formulating the rationale behind the feature interpretation. All descriptions were documented in short, written form. Notes about the interpretation process as a whole, investigated tiles, comments, thoughts, ideas, etc. were recorded in a general documentation diary.

### Ground‐observation

The ground‐observation can be regarded as a targeted, LiDAR‐guided archaeological prospection and a test of the remote sensing interpretation (Banning, 2002, pp. 28–29). Based on the features mapped in the LiDAR interpretation, a survey can be planned and carried out very efficiently (Figure 5). If for time or cost constraints it is not possible to visit every single feature, a sample has to be chosen in consideration with the research aims.

In the case of the Kolophon survey, the time for field walking was limited by official survey permits. Since our research goal was the urban development of the city, priority was given to the exploration of the burial grounds and possible other monuments in their vicinity. For the field survey, the LiDAR visualizations were converted to a Mobile Atlas for display on a tablet. Both interpreted and blank visualizations were used to also allow a ‘clean’ view of the LiDAR in the field. The interpretation and mapping was carried out in ArcGis and exported to high‐resolution raster images. These were converted to a Galileo sqlite Mobile Atlas with the MapC2MapC software (http://www.the‐thorns.org.uk/mapping). For offline display and mapping on the tablet, the excellent Locus Pro mapping software (http://www.locusmap.eu) was used, which allows for quick changes between views of the LiDAR visualizations, topographical and geological maps, as well as orthophotos. The tablets used were an ASUS ME302C and a Nexus 7 (2013, ASUS‐1A019A), which both combined excellent display and sufficient memory with battery capacity for a full day‐long survey. Furthermore, we employed an external global positioning system (GPS) receiver to improve the location accuracy and battery lifetime, but the positioning and mapping had to be corrected with the LiDAR data. This combination of tablets with built‐in GPS receivers and mapping software proved highly useful and effective.

The field campaign in Kolophon took place during two weeks in September 2014. Features were targeted and approached by GPS and, if confirmed by the ground‐observation, documented as archaeological objects. The immediate area around the features was searched for other objects, signs of human activity and archaeological finds. The documentation of features during the ground‐observation was similar to their documentation in the LiDAR data and included their properties, in particular their size, shape, context and interpretation (Figure 5). The same procedure was carried out also in cases where the LiDAR data suggested the existence of an object, but the ground‐observation proved inconclusive. LiDAR interpretation is a prospection method with an independent argumentative power (Doneus and Kühtreiber, 2013, pp. 38–39): if the rationale of a LiDAR interpretation was considered to be convincing, a feature was marked and mapped as a possible object, even if it was unambiguously recognizable in the field.

After each day of survey activity, the GIS‐database was updated with the results and the scan data was re‐examined against the background of the newly available information. This sometimes led to the discovery of features missed in the first interpretation and a continuously improved understanding of the LiDAR data. The recorded GPS tracks of the survey team were used to verify that no features had been missed during the ground‐observation.

During the two‐week survey, a team of two to three persons checked a total of 220 features and the larger surrounding area to test the accuracy of the scan (Table 2). Of the 220 features, 131 features were addressed as potential archaeological objects in the LiDAR interpretation. The ground‐observation supported this in 89 cases, while in 42 a natural origin was considered more likely. Thus, 89 features were marked as natural during the remote sensing. In 71 cases this interpretation was confirmed, while 18 were readdressed as archaeological objects after the ground‐observation. An additional 53 objects were documented that were not marked in the LiDAR interpretation at all. They mostly included archaeological objects with very low surface reliefs, such as graves or burial mounds consisting of simple stone settings (Table 2). In some cases, features that had been addressed as a singular object turned out to consist of several different structures, e.g. a burial mound and an adjacent, but unconnected terrace wall.

**Table 2:** Results of LiDAR interpretation and ground‐observation in the Kolophon survey.

Interpretation	Total	Supported by ground‐observation		Refuted after ground‐observation		
Archaeological features	131	89		42		
Natural features	89	71		18		
—	Burial mound	Grave terrace	Grave	Wall/structure	Other	Total
LiDAR interpretation + ground‐observation	73	5	8	8	13	107
Ground‐observation only	22	5	10	9	7	53
Total	95	10	18	17	20	160

### Data integration

While the LiDAR interpretation itself already provides an abundance of archaeological information, it becomes particularly useful if combined with other sources of data, like aerial photography, LiDAR data, field surveys, geophysics, historical maps, geological maps, travel reports and archaeological documentation (Campana, 2008; Opitz, 2009).

**Figure 6:**
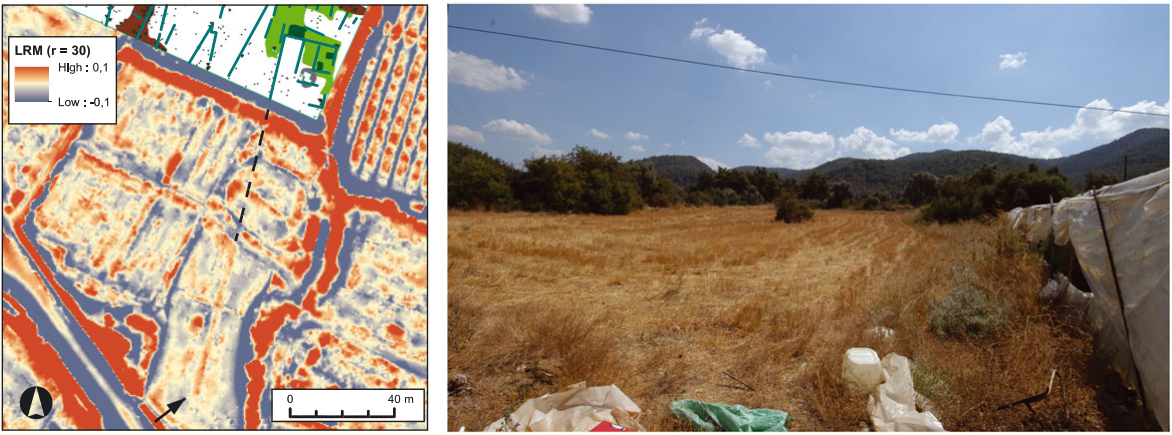
(Left) Ground penetration radar (GPR) interpretation, conducted by Posselt&Zickgraf Prospektionen, and local relief model (LRM) visualization (*r* = 30, histogram stretch = ˗0.1/0.1). The walls identified in the GPR correspond in their alignment to a similar structure visible as a slight elevation of only a few centimetres to the south. (Right) Photograph of the field from the southeast corner. [Colour figure can be viewed at wileyonlinelibrary.com]

In areas of modern agricultural activity, surface alterations are frequent and overlap each other to an extent that make a refined interpretation nearly impossible. On a field within the city walls of Kolophon a 58 m long, straight elevation was identified in the LRM visualization, but was not visible during ground‐observation due to its small height (Figure 6). Similar features exist on many fields and commonly result from modern leveling, construction, or agricultural activity (e.g. drainage). However, we also carried out geophysical prospection within the city as part of the survey project. In a field 60 m north of the LiDAR feature, GPR data shows wall structures which, in combination with the pottery survey, are probably related with the reorganization of the city during the fourth century (Gassner *et al*., 2017). The LiDAR feature in Figure 6 shares orientation and alignment with these walls and may represent an analogue structure.

**Figure 7:**
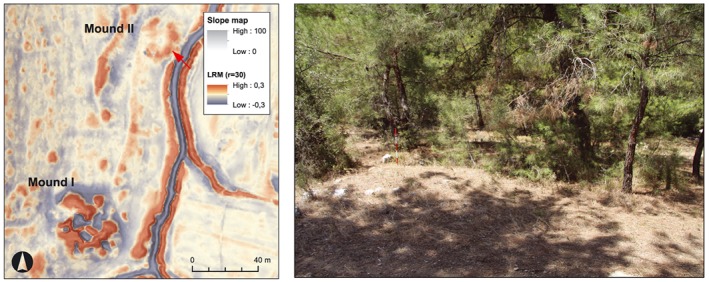
(Left) Valley cemetery in local relief model (LRM) visualization (*r* = 30, histogram stretch = ˗0.3/0.3), combined with 50% opacity slope map (histogram stretch = standard deviations). The heavily disturbed, but still several metres high geometric burial Mound I is easily recognizable in the terrain, while Mound II is slightly visible only in the LRM visualization. (Right) Photograph of Mound II from the northeast, looking southwest, with the mound visible as a slight elevation. [Colour figure can be viewed at wileyonlinelibrary.com]

The strength of the LRM visualization was further shown during the investigation of burial mounds in the geometric necropolis of Kolophon (Figure 7). One of the burial mounds, Mound II, was excavated by the American expedition in 1922. It was described as a circular mound of about 20 m in diameter and 1–2 m in height. Its location was known in relation to the particularly prominent and large Mound I, which had been successfully located in 2010 (Mariaud, 2011; Gassner *et al*., 2017). At the described distance and direction, we discovered illicit excavations, but no significant archaeological remains. While no mound was discernible with the naked eye, the LRM visualization clearly showed a round structure at this location (Figure 7). This elevation of 20 m in diameter was however only preserved to a height of about 10 to 20 cm. Both the rediscovery of the mound and the recognition of the LiDAR feature as such relied on the integration of the excavation diaries of 1922 with the LiDAR scan conducted in 2013.

## Discussion

### Evaluation of results

#### Success rate, obtrusiveness and visibility

The relatively high number of false‐positive results (Table 2) can be attributed to our aim of using the interpretation as a guide for a thorough ground‐observation. A more conservative interpretation would have yielded a higher percentage of correct interpretations, at the cost of altogether missing objects.

The vast majority of objects identified in the LiDAR interpretation were burial mounds and grave terraces (Table 2). This confirmed an expected bias of the LiDAR interpretation towards objects of larger size. Features with very low surface relief often did not show a noticeable contrast in the LiDAR data and were only discovered during the ground‐observation. Illicit excavations proved to be a highly reliable indicator for archaeological objects and were often the only means by which smaller graves could be identified. Almost all confirmed features were situated in forested or overgrown areas. A considerable number were thickly covered by scrub in barely accessible areas. They would most likely not have been found by traditional field‐walking. Additionally, documented features comprise pathways, river courses, geological phenomena and modern buildings and agricultural activity. These were not recorded as archaeological objects, but their documentation greatly supported a contextual understanding of the landscape. Although their general course was already known (Schuchhardt, 1886; Bruns‐Özgan *et al.*, 2011), the LiDAR data also proved helpful in documenting the exact course of the city walls in difficult and overgrown terrain.

During the ground‐observation it quickly became evident that feature preservation and discernibility in LiDAR data were mostly linked to feature size and modern land use. The landscape palimpsest shows most clearly the recent and heaviest inscriptions; older features are only visible if later formation processes have not destroyed them. This is seldom the case in areas suitable for cultivation, although large objects such as city walls obviously stand a better chance of survival. In forested and pasture areas, surface features are much better preserved and consequently detectable in LiDAR data. Knowledge of local agricultural and landscaping activities proved to be very important for the correct interpretation of the landscape palimpsest. In the case of the Kolophon survey, forested areas were mainly restricted to mountainous areas. In the rugged terrain it is difficult to distinguish archaeological features from the surrounding surface. This situation can be improved by first‐hand knowledge of the terrain and the cooperation with a geologist or geoarchaeologist during the interpretation and survey. In any case, the results and experiences from ground‐observations should continuously contribute to the interpretation process.

**Figure 8:**
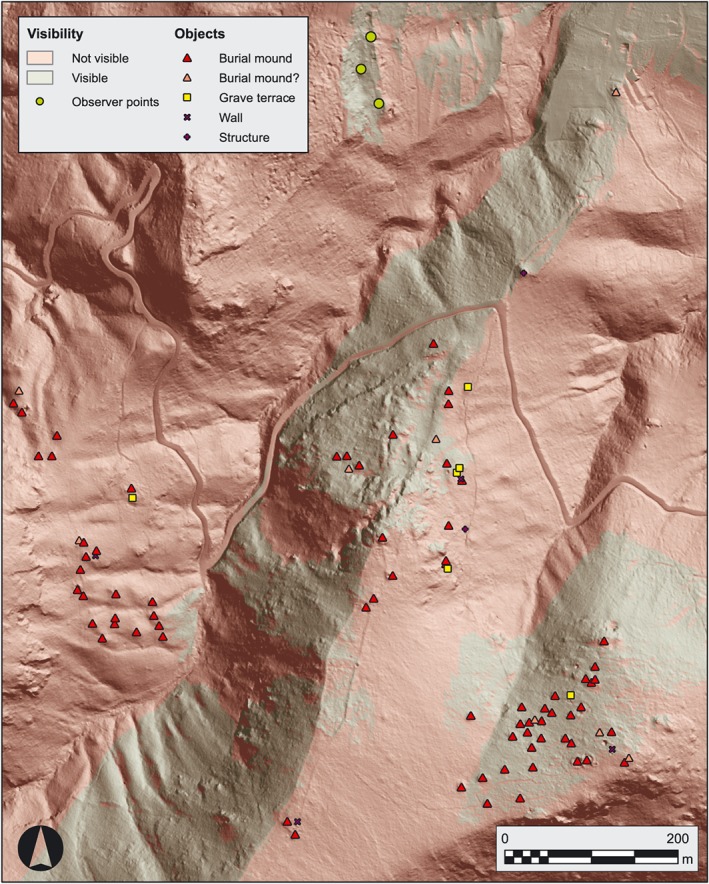
Acropolis, south necropolis and southwest necropolis of Kolophon in LiDAR hillshade. The viewshed assumes observer points [height (*h*) = 1.5 m] on the terrace of the city's only known intra‐urban sanctuary, the Metroon on the Acropolis. Areas visible from at least one point are marked green. The Metroon terrace extended further to the northeast in antiquity and the viewshed assumes no vegetation, but nonetheless burial mounds within the south necropolis are noticeably confined to areas visible from the Metroon, while those of the southwest necropolis are not. The burial mounds along the ridge were most likely visible as well, as they are heavily eroded today. It is unclear if the city wall was high enough in antiquity to impede the field of vision from the Metroon. [Colour figure can be viewed at wileyonlinelibrary.com]

Several venues for further research were suggested by the results of the LiDAR interpretation and the guided survey. The role visibility played for the installation of some of the burial mounds became apparent (Figure 8) and the pathways connecting the burial grounds to the landscape are readily observable. The aspects of visibility and mobility within the funerary landscape become considerably more accessible by the means of a LiDAR perspective of the landscape.

#### Chronology

A major limitation of LiDAR interpretations is the inability to date surface features directly. The age of features can be estimated from:

- Typological comparison with dated objects
- Spatial association with dated objects
- Cross‐cutting relationships
- Modern features can often be identified in the aerial photography

With the exception of objects that are evidently related to modern activities, a ground‐observation is necessary to collect dating evidence. Besides general typological considerations, the dating will rest on small finds collected in the vicinity of mapped objects. The dating of objects by surface finds faces several methodological issues (Banning, 2002, pp. 204–206). Besides the quantification of the find material (Mattingly, 2000, pp. 11–12), the dating is based on the evaluation of the stratigraphic connection of the surface finds with the associated objects (Doneus, 2013a, pp. 147–150). Depending on formation processes, the quality of the dating evidence can vary considerably.

The number of find materials from the ground‐observation in Kolophon was extremely low and commonly limited to surface finds in the dump material of illicit excavations. These finds, especially those with a clear stratigraphic connection of the dump material with the features, provided quite reliable, but very punctual chronological insights. The majority of the surface objects were dated by typological comparisons and spatial association, which only allows for rough dating estimates. Consequently, LiDAR data are very well suited for establishing the presence or absence of archaeological features, provided any relief is preserved. Even in combination with ground‐observation, not all features can be dated precisely, and LiDAR data often cannot provide insight into specific time‐periods.

### Comparison of LiDAR data visualizations

**Figure 9:**
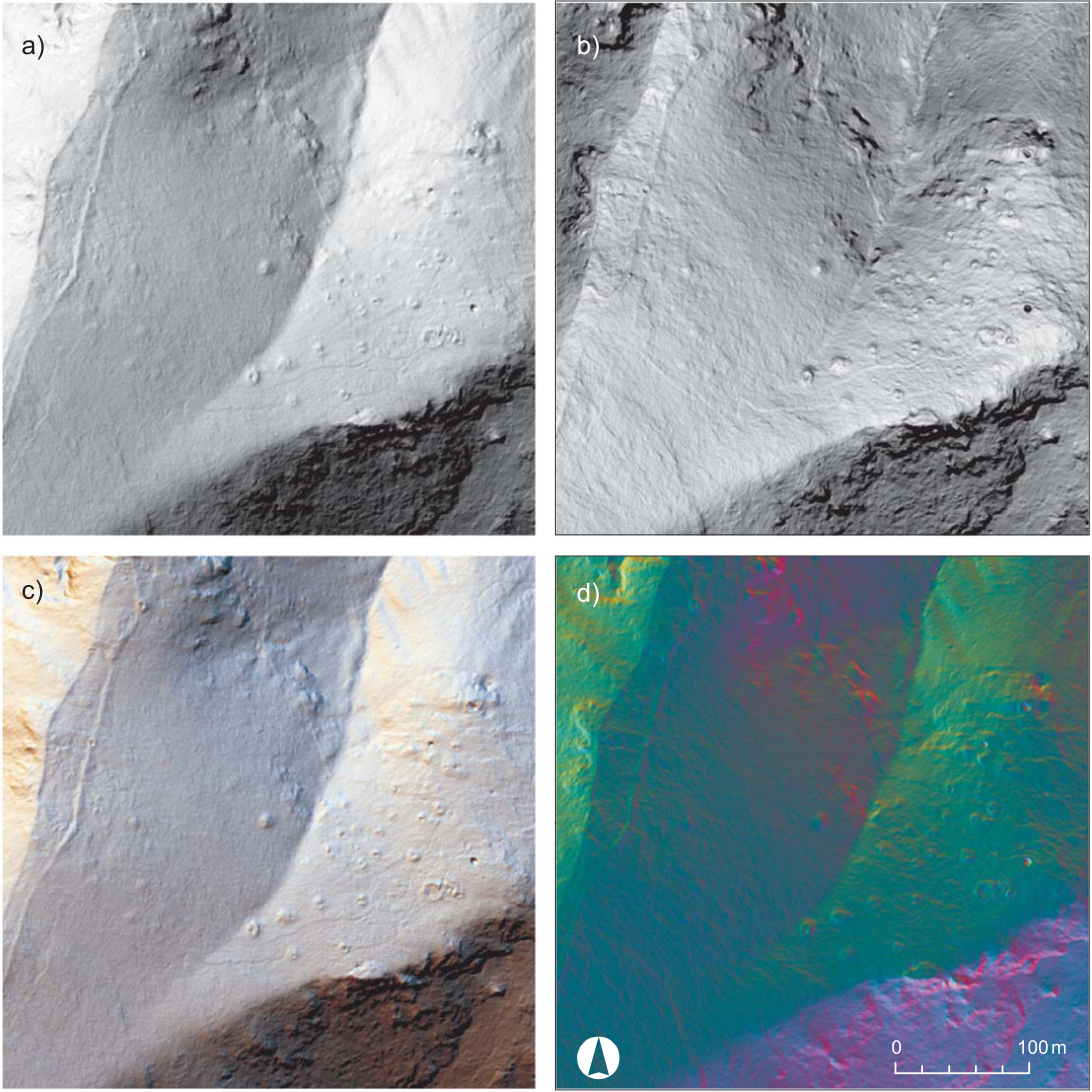
South necropolis of Kolophon on Taşyalak Tepesi. (a) Hillshade (*a* = 315, *e* = 35, histogram stretch = standard deviation); (b) slope map (histogram stretch = standard deviation); (c) multishade (*d* = 16, *e* = 35, histogram stretch = standard deviations); (d) principal component analysis (composite image, *d* = 16, *e* = 35; histogram stretch = standard deviations). The large burial mounds are easily identifiable in all visualizations, while the visibility of pathways differs notably. [Colour figure can be viewed at wileyonlinelibrary.com]

Hillshades and slope maps (Figure 9) are a commonly used visualization of relief data (Kokalj *et al.*, 2013, pp. 103–105). Basic hillshade visualizations have been criticized for their limitations in archaeological interpretation of LiDAR data (Challis *et al.*, 2011, 287; Hesse, 2010, 2012). A major concern is that features aligned parallel with the direction of illumination are not visible (Devereux *et al.*, 2008, pp. 470–471; Figure 9a). Further, the recognition of features in areas that are too exposed to the illumination or too hidden from it is difficult and requires readjusting of the histogram stretch. These problems can be avoided by creating several hillshades illuminated from different angles and directions, but this is both time and labour consuming. Multishades combine hillshades from several directions, but tend to reduce contrast differences, making individual features less distinguishable (Figure 9c). A PCA condenses the information of multiple hillshades, but can be considerably more difficult to understand intuitively (Devereux *et al.*, 2008; Štular *et al*., 2012, p. 3355; Figure 9d). Slope visualizations offer a relatively straightforward representation of the steepness of terrain, which makes it easy to recognize steep changes of height (Figure 9b).

Despite their shortcomings, hillshades are the most readable visualization of digital elevation data (Doneus, 2013a, p. 217). The data representation is intuitively understandable and features can easily be related to the objects they represent. Using two hillshades with different illumination directions was a viable, low‐effort starting point for our interpretation, especially when initially working on a larger scale. Slope maps proved similarly easy to understand and were extensively used in this stage. Another advantage was that both hillshade and slope map visualizations are included in ArcGIS and did not require the use of additional software.

**Figure 10:**
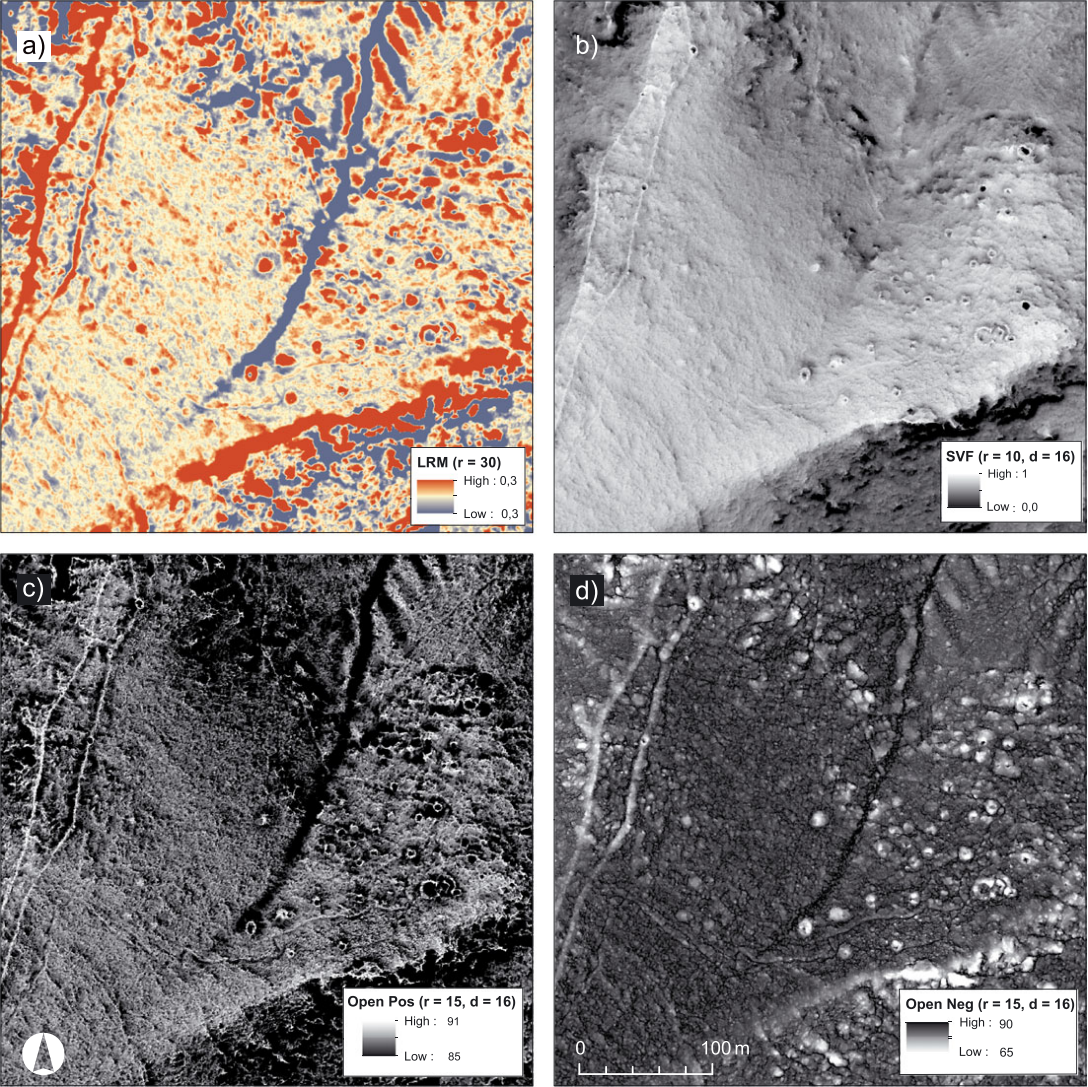
South necropolis of Kolophon on Taşyalak Tepesi in (a) local relief model (LRM) (*r* = 30, histogram stretch = ˗0.3/0.3); (b) sky‐view factor (SVF) (*r* = 10, *d* = 16, histogram stretch =0.7/1); (c) positive openness (*r* = 15, *d* = 16, histogram stretch =85/90); (d) negative openness (*r* = 15, *d* = 16, histogram stretch =65/90). [Colour figure can be viewed at wileyonlinelibrary.com]

LRMs (Figure 10a) are abstract visualizations developed for the recognition of small‐scale topographic features (Figure 10a; Hesse, 2010, 2012). A low‐pass filter is applied to the LiDAR DTM to create a smoothed elevation model containing the large‐scale topographic features of the terrain, which is subsequently subtracted from the original high‐resolution DTM. This visualization is particularly useful for archaeological interpretations concerned with small‐scale height differences. The kernel size of the low‐pass filter determines the degree of terrain smoothing and needs to be chosen according to the size of the expected archaeological objects. Recommended values range from 15 to 30 m; larger archaeological features are rare and will most likely be recognizable in any visualization, while smaller features remain unrecognizable unless they are situated on very convex or concave terrain (Hesse, 2010, p. 71).

LRM visualizations create very complex impressions of the micro‐topography due to their abstract perception and high sensitivity to height variations. They tend to create visual artefacts and misrepresent the size of features on slopes and in irregular terrain (Doneus, 2013b, pp. 6436–6437). Without the metadata of the search radius, the search directions and the histogram stretch, it is hard to read the data visualization. Constant cross checking of inspected features in other visualizations is generally recommended, but in particular so for the LRM visualization, as the understanding of the topography is challenging. It proved highly useful to overlay the visualizations with a 50% transparent slope map to mitigate the drawbacks of the abstract terrain representation (Figure 10a). LRM is a powerful approach for identifying small, low and weakly defined features. During the advanced stages of our interpretation, the LRM and slope map combination was the preferred method for a detailed interpretation.

Openness (Figures 10b, 10c, 10d) of LiDAR DTMs is determined as the mean value of the largest possible zenith (positive openness, Figure 10c) or nadir (negative openness, Figure 10d) of profiles drawn in eight or more directions from any given point (Doneus, 2013b). The length of this profile needs to be determined according to the research question and the size of features to be identified. Recommended values range from 5 to 30 m (Doneus, 2013b, p. 6429). Openness is very similar to the SVF visualization (Figure 10b), which also measures the zenith angle of a given point in several directions, but only above the horizontal plane. Therefore the slope of the general terrain is visible in the SVF visualization, while it is not represented in the openness visualization. Similarly to the LRM visualization, the removal of the general topography makes openness a highly abstract representation of the terrain. It does however offer the advantage that similar surface structures will be displayed in the same way, regardless of the slope of the underlying macro‐topography (Doneus, 2013b, p. 6436). Burial mounds on sloping surfaces will show the same data value as those on horizontal surfaces. Openness offers a very clear distinction of changes in micro‐relief and marked slope breaks, such as the ridges of paths, riverbeds, terraces and burial mounds. While positive openness highlights the upper edges of slope changes, negative openness highlights lower edges. Negative openness allows a very accurate tracing of concave features, while positive openness is best used for documenting convex features (Doneus, 2013b, pp. 6432–6433, 6436). In our study at Kolophon, openness was found to be hard to use for interpretation, as the very abstract terrain representation made an immediate understanding of the data challenging.

#### Image enhancements and colour gradients

**Figure 11:**
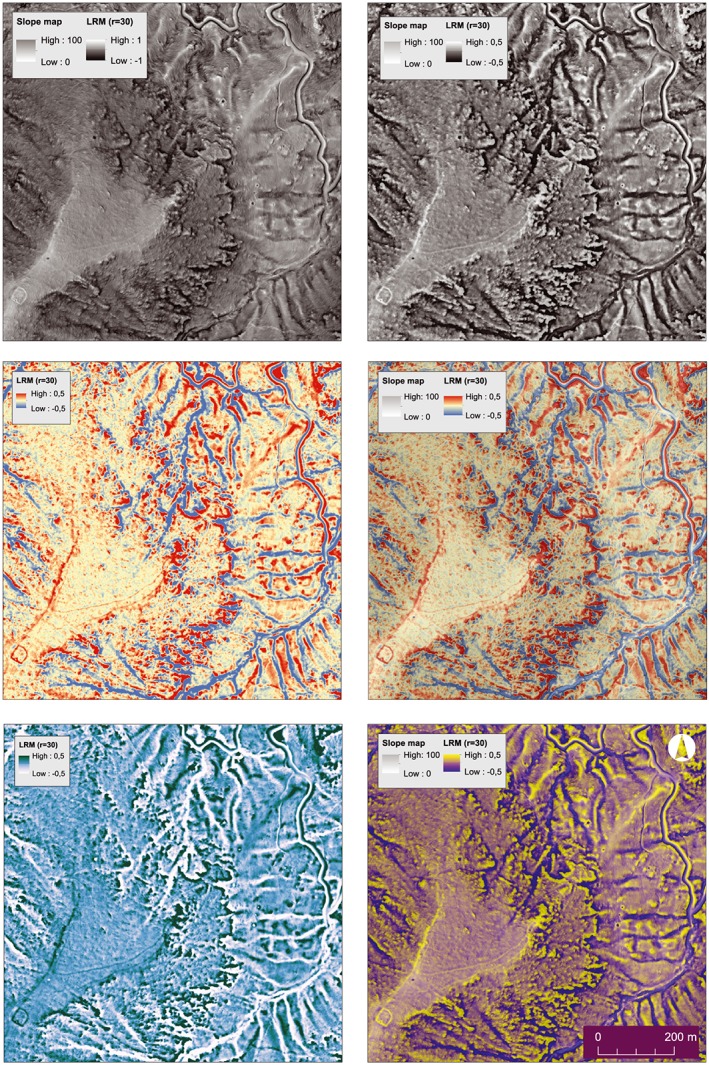
South necropolis of Kolophon on Taşyalak Tepesi. Local relief model (LRM) visualization (*r* = 30) in different colour gradients and with different histogram stretches. Confirmed objects in the area are several burial mounds, illicit excavations, paths, a compound, and a wall‐like structure confining the ridge of the hill. [Colour figure can be viewed at wileyonlinelibrary.com]

All visualizations were subject to image enhancement by histogram stretch. Depending on the selected parameters for image enhancement and colour gradients, the same datasets can be displayed quite differently (Figure 11). These parameters are a part of the metadata of the interpretation (Kokalj *et al.*, 2013, pp. 105–107). Variations of the minimum and maximum value thresholds of the colour gradients can enhance contrasts, making even minimal height differences visible. During the interpretation process the thresholds of the histogram stretch were often adapted to the scale of the specific interpretation.

Most programs display LiDAR visualizations in black and white gradients by default. Due to the limitations of the human eye in distinguishing between different shades of grey, coloured visualizations allow for a better recognition of contrast variations (Girard and Girard, 2003, pp. 57–58). The high contrast of complementary colours (yellow/red/blue, red/blue, yellow/purple, red/green, orange/blue) can be used to this effect, while a slight reduction of the saturation of complementary colours can reduce distortion due to the different optical weight of the colours (Samsel *et al.*, 2014). Ultimately, perception of visualizations depends on viewing habits and personal preference. The authors found black and white gradients to offer the most comprehensible view of the data (Figure 11). Red, yellow and blue colour gradients made it easier to estimate height differences, while gradients of two complementary colours enhance contrasts noticeably – at the expense of lucidity.

### Discussion of the interpretation process

The LiDAR‐based perspective on landscapes gives a non‐selective view of the ground: Without interpretation, the DTM shows an omnium‐gatherum of various structures, objects, places, paths, etc. embedded in the physical landscapes (Mlekuž, 2013a, pp. 93–94). Research into a semi‐automatic detection of LiDAR features is ongoing (e.g. Sevara *et al.*, 2016) and will certainly supplement the interpretation process in the near future. Nevertheless, to reach a meaningful archaeological interpretation, human agency is required.

**Figure 12:**
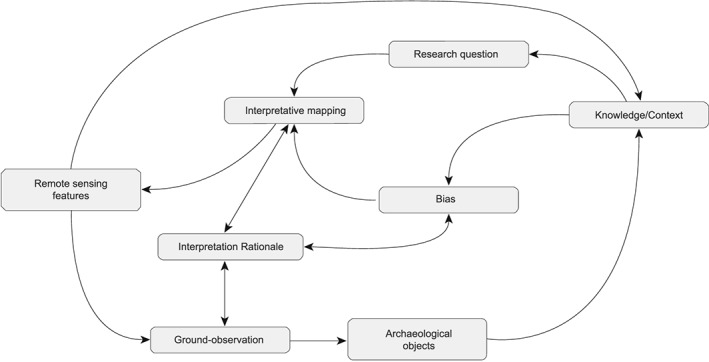
Interpretation process as a simplified flow chart.

Interpretation of LiDAR data is strongly influenced by archaeological/environmental knowledge and experience in handling LiDAR data (Doneus and Kühtreiber, 2013; Palmer, 2013, pp. 78–81; Cowley, 2013). Experience and context will guide the identification of features, but also change constantly during the interpretation process due to insights derived from the interpretation itself (Figure 12). The interpretation of remote sensing data has been described as a hermeneutic approach (Brophy, 2005; Doneus, 2013a, pp. 196–197).

The applicability of the hermeneutic process to human action and understanding has been discussed extensively in philosophical literature. Ricœur (1981, pp. 203–218) argues that all phenomena that can be objectified, like a text, can be understood in a similar fashion. Following this idea, archaeologists have applied hermeneutic principles to the understanding of material culture (Moore, 1990, pp. 97–99, 111–112; Tilley, 1991, pp. 115–118; Hodder, 1999, pp. 21–79). The intention of any hermeneutic interpretation can be seen in exemplifying the reference function of the ‘text’ to the world (Mattern, 1996, p. 112). For our purposes, this corresponds with exemplifying the meaning of the landscape topography, as represented in the LiDAR DTM surface, in relation to our research interest. A useful analogy is to understand a landscape as a palimpsest: traces of activities are continually inscribed into it, overlapping and erasing earlier inscriptions (Crawford, 1953, pp. 51–52; Bailey, 2007, pp. 123–124, 203–210; Mlekuž, 2013b). The analogy is not only apt for describing the spatial and temporal dimensions of a landscape and the character of its features, but also for the process of understanding LiDAR data: it can be described as an attempt to read and understand the landscape palimpsest.

Interpretation is inherently subjective and it is impossible to completely distance oneself from preconceived opinions (Gadamer, 2010, pp. 270–290). Openness towards the opinion of the text and recognition of the interpreter's preconceived opinions is therefore necessary: Understanding is acquired by evaluating the text against these preconceived opinions (Gadamer, 2010, pp. 253–254, 273–274). Ricœur (1969, pp. 33–49) further distinguishes between two conflicting approaches, the hermeneutics of trust and suspicion. Hermeneutic attempts to enlarge meaning and understanding can be juxtaposed with reductionist attempts, which distrust the meaning of symbols and their understanding (Mattern, 1996, pp. 58–64). The latter operates with explanatory methods more commonly associated with natural sciences and structuralism. Both approaches are not mutually exclusive. For Ricœur (1969, pp. 68–70), meaning is derived from the dialectic between the hermeneutics of trust and suspicion, of understanding and explanation.

In remote sensing the recognition of contrasts, shapes and patterns is the subjective part of interpretation, guided by preconceived opinions, knowledge, and expectation. Identified features and their relationship to the whole are used in an attempt to enlarge the meaning of the LiDAR palimpsest. At the same time, the interpretation consists of the definition, description and analysis of structural units and their relationships with each other. During this part of the process, a distrustful approach to ones identifications and the meaning derived from the interpretation is advisable. Enlarging and distrustful approaches to interpretation supplement each other in the attempt to successfully read the individual features of LiDAR data as a coherent text, embedded in an archaeological context. Interpretation conceives itself in the form of a hermeneutic spiral, a constant back and forth between the understanding of the individual parts and the whole, between symbols, sentences, and the text, between enlargement and distrust of meaning. By defining criteria for interpretation and formulating a rationale based on these criteria, both approaches can be objectified.

This objectification is necessary, as interpretations cannot be verified or disproven by empirical studies (Hodder, 2012, pp. 22–23). The ground‐observation itself contains elements of interpretation and it is guided or influenced by knowledge generated during remote sensing interpretation. Ground‐observation cannot verify an interpretation, even if it would cover all the features identified in the remote sensing, but rather adds a different line of argument to the interpretation (Doneus and Kühtreiber, 2013, p. 39). Even though interpretations are not strictly verifiable or falsifiable, they are not all equally valid. With the hermeneutic framework their cogency requires consistent, logical rationales based on the original object of investigation in relation to the theoretical framework of the specific study (Moore, 1990, pp. 95–96).

Unlike an actual text, the LiDAR data as the original object is usually not available for others as a reference. LiDAR interpretation shares this problem with other archaeological methods [e.g. remote sensing, excavation and survey documentation (Hodder, 1999, pp. 30–31)], but it is amplified by the complex visualization processes involved in the interpretation. This is a major methodological issue and challenges the optimism that the growth of archaeological facts will necessarily increase our understanding of the past (c.f. Trigger, 2006, pp. 17–26, 38–39, 531). As it is not possible for others to read the original ‘text’ or evaluate the metadata and subjective interpretation factors of earlier studies, there is an acute danger of producing datasets which grow beyond the capabilities of archaeologists to integrate. There are laudable efforts to improve this situation by making LiDAR visualizations available via WebGIS applications (e.g. Schwerin *et al.*, 2016), but this is not yet considered the best practice approach for LiDAR studies.

There is no objective end to an interpretation process, since the possible contexts are unlimited. As the subjective factors of the interpretation cannot be negated completely, there are no objectively right or wrong remote sensing interpretations, only more or less convincing ones (Palmer, 1989, p. 56; Doneus, 2013a, pp. 204–205). Therefore, an interpretation should never be considered finished (Doneus and Kühtreiber, 2013, p. 47). It can constantly be revised and improved, but usually the end will be determined by practical reasons such as funding and time‐constraints.

#### Fallacies and interpretative bias (Figure 13)

**Figure 13:**
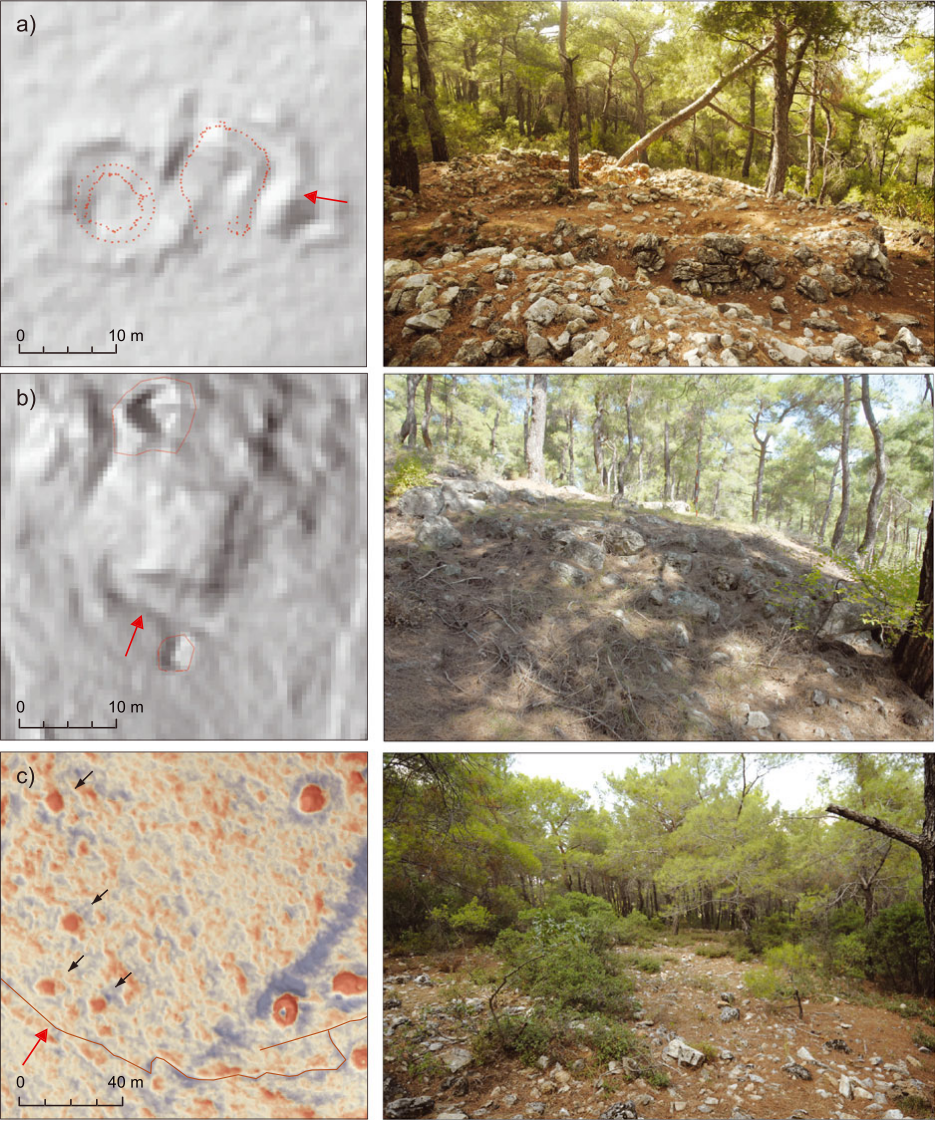
Bias and limitations of LiDAR interpretation, with LiDAR view on the left and a photograph from the ground‐observation on the right. Red arrows indicate the camera view. (a) Hillshade (*a* = 315, *h* = 35) and total station measurements of burial mound walls in Kolophon's south necropolis. While the outline of the eastern burial mound was partially captured by the LiDAR scan, the western burial mound is missing altogether. (b) Slope map and LiDAR interpretation before ground‐observation in the southwest necropolis. In the centre of the image, a large rectangular grave terrace is easily distinguishable, but was missed during the interpretation. (c) Local relief model (LRM) visualization (*r* = 30, histogram stretch = ˗0.3/0.3) and slope map (50% opacity, histogram stretch = standard deviations) of actual burial mounds to the east and accumulations of rubble stones (karst) to the west. [Colour figure can be viewed at wileyonlinelibrary.com]

The hermeneutic approach helps to diminish personal bias (Brophy, 2005, p. 7; Doneus, 2013a, p. 197). Nevertheless, fallacies may enter the interpretation process anytime:

- The DTM surface suggests a contemporaneity of features. These palimpsests were however, inscribed successively into a landscape that originally may have looked different from the present‐day surface. Likewise, the lack of vegetation in the LiDAR DTM can suggest visual or physical relationships that did not necessarily exist in the past due to different vegetation cover, changes in topography or by structures that no longer exist. This may seem trivial, but it can take considerable effort to not automatically equate spatial and chronological association. Overlaying chronological information over the LiDAR landscape can help, but will likewise influence the interpretation
- The high resolution of the topography gives the impression of a ‘real’ terrain surface, while it is actually just an artificially created, detailed representation and the result of more or less accurate measurements and filtering processes. During the interpretation, one tends to ignore issues of scan resolution, filtering algorithms and interpolation (Opitz, 2013). This is a particular issue with DTMs created for non‐archaeological purposes. Even in the case of the scan data in Kolophon (resolution of 0.5 m, high accuracy), short or thin walls and smaller illicit excavations were missed or misrepresented in the scan data (Figure 13a).
- The archaeological knowledge of the interpreter leads to an interpretive bias. We expected that the majority of visible features in Kolophon were round burial mounds. While this proved to be largely correct, during the ground‐observation it turned out that a number of the identified ‘burial mounds’ were actually grave terraces. Grave terraces were unrecognized during previous surveys and subsequently in the LiDAR interpretation, even when some are fairly evident due to their rectangular shape and clear boundaries (Figure 13b). In other cases, eroded or disturbed terraces were misinterpreted as burial mounds (Figure 5). There is a constant danger of finding what one expects.
- The criterion of *association* brings with it that higher numbers of potential features are mapped in specific areas of archaeological interest. This introduces a risk of interpretative bias towards clusters of features, while singular and remote features will be overlooked. This situation could be avoided by selecting a stratified sample of features for ground‐observation during the survey (Banning, 2002, pp. 155–156). Similarly, over‐interpretation and circular arguments are introduced if features are identified in relation to other remote sensing features. For this reason, a cluster of small, round elevations in the west of the south necropolis was misidentified as a group of burial mounds since they seemed to be associated with the path towards the necropolis (Figure 13c). The ground‐observation indicated that these elevations are simply related to the topography of the rock outcrop there.

Besides various factors like knowledge, experience, (selective) interest, motivation, etc., interpretation is also strongly influenced by the personality of the interpreter concerning over‐ and under‐interpretation of the data (Brophy and Cowley, 2005, p. 22). Reproducible documentation, ground‐observation and the inclusion of multiple persons in the interpretation/observation process are advisable to minimize misinterpretation and bias.

## Conclusions

LiDAR in combination with orthophotos is extremely useful for the detection of archaeological remains below Mediterranean evergreen vegetation and/or dense maquis. In areas where the surface relief of archaeological remains is well preserved, LiDAR data is not only a powerful tool for very time‐effective archaeological mapping and targeted field‐surveys, but also for the documentation and topographical contextualization of archaeological and natural features.

Earth's surface is shaped by a plethora of natural and anthropogenic processes that LiDAR data makes unselectively visible. Therefore, a reasonable interpretation of LiDAR topographic data demands a critical evaluation of all assorted metadata and a close cooperation of interpreters with archaeological and geological backgrounds. An awareness of the hermeneutic approach is required to contain the inherent, subjective bias of the interpretation. A detailed description and documentation of the interpretation process will help readers, particularly those not familiar with remote sensing or LiDAR visualizations, to assess and evaluate the results.

Good interpretative results could already be achieved using the basic hillshade and slope visualizations. Despite their shortcomings, we recommended them as a starting point for an interpretation, in particular for users inexperienced with LiDAR data. The application of additional visualizations depends on the research interest, but within the Kolophon project, LRM and openness visualizations proved most useful for purposes of detailed interpretative mapping and documentation.

LiDAR interpretation is a very potent archaeological remote sensing method that provides a unique view of the topography of a landscape. It provides the best results when it is closely tied to complementary methods, in particular ground‐observations and aerial photography, but also historical photographs, geophysics, geological and topographic maps.

## Declaration of interest statement

The authors have no conflict of interest to declare.
